# Thrombin generation and implications for hemophilia therapies: A narrative review

**DOI:** 10.1016/j.rpth.2022.100018

**Published:** 2022-12-21

**Authors:** Robert F. Sidonio, Maureane Hoffman, Gili Kenet, Yesim Dargaud

**Affiliations:** 1 Aflac Cancer and Blood Disorders Center of Children's Healthcare of Atlanta, Atlanta, Georgia, USA; 2 Department of Pediatrics, Emory University, Atlanta, Georgia, USA; 3 Department of Pathology, Duke University School of Medicine, Durham, North Carolina, USA; 4 The Israeli National Hemophilia Center and Thrombosis Unit, Sheba Medical Center, Tel Hashomer, Israel; 5 The Amalia Biron Thrombosis Research Institute, Tel Aviv University, Tel Aviv, Israel; 6 Unité d’Hémostase Clinique, Centre National de Reference de l'Hemophilie, Hôpital Cardiologique Louis Pradel, Université Lyon, Lyon, France

**Keywords:** antithrombins, blood coagulation tests, fitusiran, hemostasis, hemophilia, therapies

## Abstract

Thrombin plays an essential role in achieving and maintaining effective hemostasis and stable clot formation. In people with hemophilia, deficiency of procoagulant factor (F)VIII or FIX results in insufficient thrombin generation, leading to reduced clot stability and various bleeding manifestations. A correlation has been found between the bleeding phenotype of people with hemophilia and the extent of thrombin generation, with individuals with increased thrombin generation being protected from bleeding and those with lower thrombin generation having increased bleeding tendency. The amount, location, and timing of thrombin generation have been found to affect the formation and stability of the resulting clot. The goal of all therapies for hemophilia is to enhance the generation of thrombin with the aim of restoring effective hemostasis and preventing or controlling bleeding; current treatment approaches rely on either replacing or mimicking the missing procoagulant (ie, FVIII or FIX) or rebalancing hemostasis through lowering natural anticoagulants, such as antithrombin. Global coagulation assays, such as the thrombin generation assay, may help guide the overall management of hemostasis by measuring and monitoring the hemostatic potential of patients and, thus, assessing the efficacy of treatment in people with hemophilia. Nevertheless, standardization of the thrombin generation assay is needed before it can be adopted in routine clinical practice.

## Essentials

• The goal of hemostasis is to generate sufficient thrombin to form a stable clot.
• Hemophilia is the result of a deficiency of factor (F)VIII or FIX, leading to reduced thrombin generation.
• Thrombin generation may be an accurate marker of bleeding tendency.
• All hemophilia treatments aim to restore thrombin generation to achieve effective hemostasis.

## Graphical abstract

**Figure:**
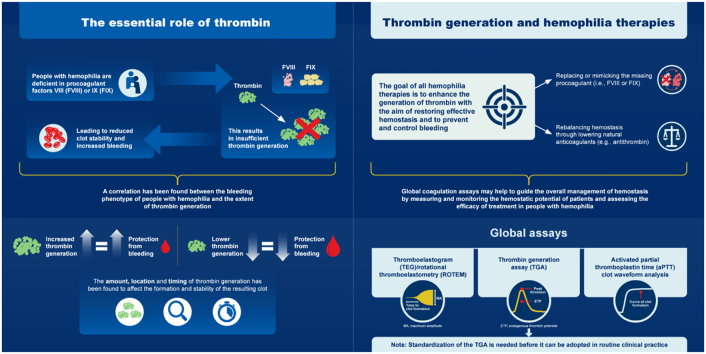

## 1 Introduction

Hemophilia stems from the deficiency of factor (F)VIII or FIX that results in insufficient thrombin generation, leading to an unstable clot and excessive bleeding [[1], [2], [3]]. The goal of all currently available and investigational hemophilia therapies, including both factor replacement and nonfactor therapies, is to restore sufficient thrombin generation to achieve effective hemostasis [4]. Nonfactor therapeutics also provide alternative strategies to improve hemostasis compared with traditional factor replacement therapies [5]. Various nonfactor therapies include an anti-FIXa/FX bispecific antibody mimicking activated FVIII (ie, emicizumab), a small interfering RNA (siRNA) to lower antithrombin levels (ie, fitusiran), antibodies that bind to tissue factor pathway inhibitor (TFPI) (eg, concizumab), or a modified serpin inhibiting activated protein C (APC) [5,6].

Hemostasis is the natural, balanced, and dynamic process of stopping bleeding at the site of injury while maintaining the integrity of blood circulation [7]. Thrombin is an enzyme central to the hemostasis, which involves a set of tightly coordinated proteolytic reactions that occur on specific cell surfaces, ultimately resulting in the formation of a stable clot [1,8]. Compared with the coagulation cascade (factor-centric) model of hemostasis, the cell-based (thrombin-centric) model emphasizes the importance of appropriately localized and regulated thrombin generation [8,9]. The amount and pattern of thrombin generation are governed by multiple components of the coagulation process, including levels of procoagulant proteins (such as FVIII and FIX) and anticoagulant proteins (such as antithrombin and TFPI) [3,9].

The purpose of this review was to examine the key role of thrombin in assessing effective hemostasis in persons with hemophilia and to evaluate the approach of factor and nonfactor replacement therapies that enhance thrombin generation to promote and achieve optimal hemostasis.

## 2 The Key Role of Thrombin in Hemostasis

**Figure 1:**
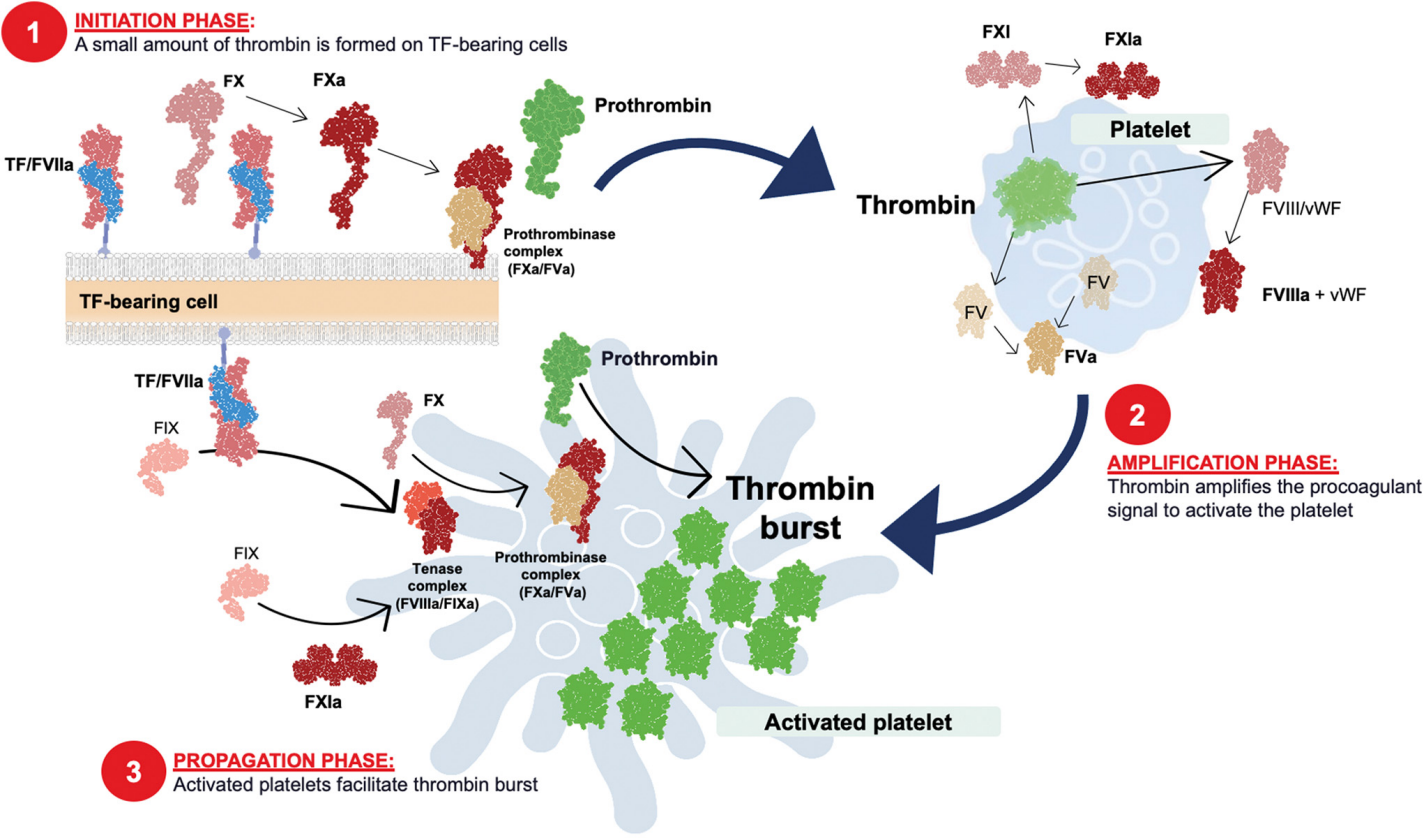
Overview of a cell-based model of coagulation: (1) initiation, (2) amplification, and (3) propagation leading to thrombin generation. FVIIIa, activated factor VIII; FXIa, activated factor XI; TF, tissue factor; VWF, von Willebrand factor

The coagulation cascade model describes the biochemical interactions of the coagulation factors involved in the hemostasis; however, the model cannot be used to explain certain aspects of bleeding and thrombosis. This led to the development of the cell-based model that goes further to explain the critical role of cells in the control of coagulation and describes the coagulation process in 3 overlapping stages: (1) initiation, (2) amplification, and (3) propagation leading to thrombin generation (Figure 1) [8,9].

Localization of procoagulant reactions on specific cell surfaces is important for the control of blood coagulation and for ensuring that the process remains at the site of injury [9]. The main cell types involved in hemostasis are tissue factor (TF)–bearing cells and platelets, both of which are involved in regulating the location, amount, and timing of thrombin generation and in the control of the coagulation process [9,10].

The overall goal of hemostasis is to generate sufficient thrombin, at the proper time and place, to form a stable clot [1]. The coagulation process is primarily initiated by TF *in vivo* [1,8,9]. During the initiation phase, small amounts of FXa, FIXa, and thrombin are produced in TF-bearing cells [1,11]. The thrombin produced during this phase is crucial for full activation of adjacent platelets into a procoagulant state [1,11]. Activated platelets expose receptors and binding sites for activated clotting factors and release partially activated forms of FV on their surface [9].

The small amounts of thrombin generated in the initiation phase allow multiple feedback loops to occur during the amplification phase. Thrombin activates cofactors V and VIII, as well as FXI, on the activated platelet surface. Activation of FVIII releases it from von Willebrand Factor [9]. This sets the stage for the large burst of thrombin production during the propagation phase.

In the propagation phase, FX is activated by tenase (FIXa/FVIIIa) complexes on the platelet surface. The resulting FXa combines with FVa to form prothrombinase (FXa/FVa) complexes on the platelet surface, which activates prothrombin (also known as FII) to produce a burst of thrombin (FIIa) generation [12].

Thrombin has numerous target substrates, but its major hemostatic function is to catalyze the conversion of soluble fibrinogen into a meshwork of insoluble fibrin strands that encase the platelet plug, stabilizing and sealing the site of injury [13]. The concentration of thrombin has been found to influence the fibrin clot structure and stability [14]; low concentrations of thrombin result in the production of thick, loosely woven fibrin strands that form a clot with low structural stability, whereas higher thrombin concentrations result in clots made up of fibrin strands that are thinner and more tightly packed and are more resistant to mechanical and enzymatic disruption [14].

The relative amounts of prothrombin and antithrombin present, as well as the levels of protein C and TFPI, are believed to be key in determining the amount of thrombin generated [1,13,15]. In bleeding disorders such as hemophilia, an imbalance between pro- and anticoagulant proteins within the hemostatic system leads to reduced thrombin generation and disrupted hemostasis. In people with hemophilia, deficiency of FVIII or FIX results in ineffective thrombin generation on the activated platelet surface during the amplification phase [16]. This results in a lack of thrombin burst and reduced clot stability, leading to various bleeding manifestations [16,17].

## 3 Thrombin Generation Correlates with Bleeding Phenotype

The hallmark of severe hemophilia is spontaneous or traumatic joint and muscle bleeding [2]. Bleeding in the joints—hemarthrosis—is a major complication in hemophilia and can lead to debilitating chronic arthropathy [16]. It usually occurs in the weight-bearing large synovial joints, which are at increased risk of trauma, such as the knees, elbows, and ankles [16].

**Figure 2:**
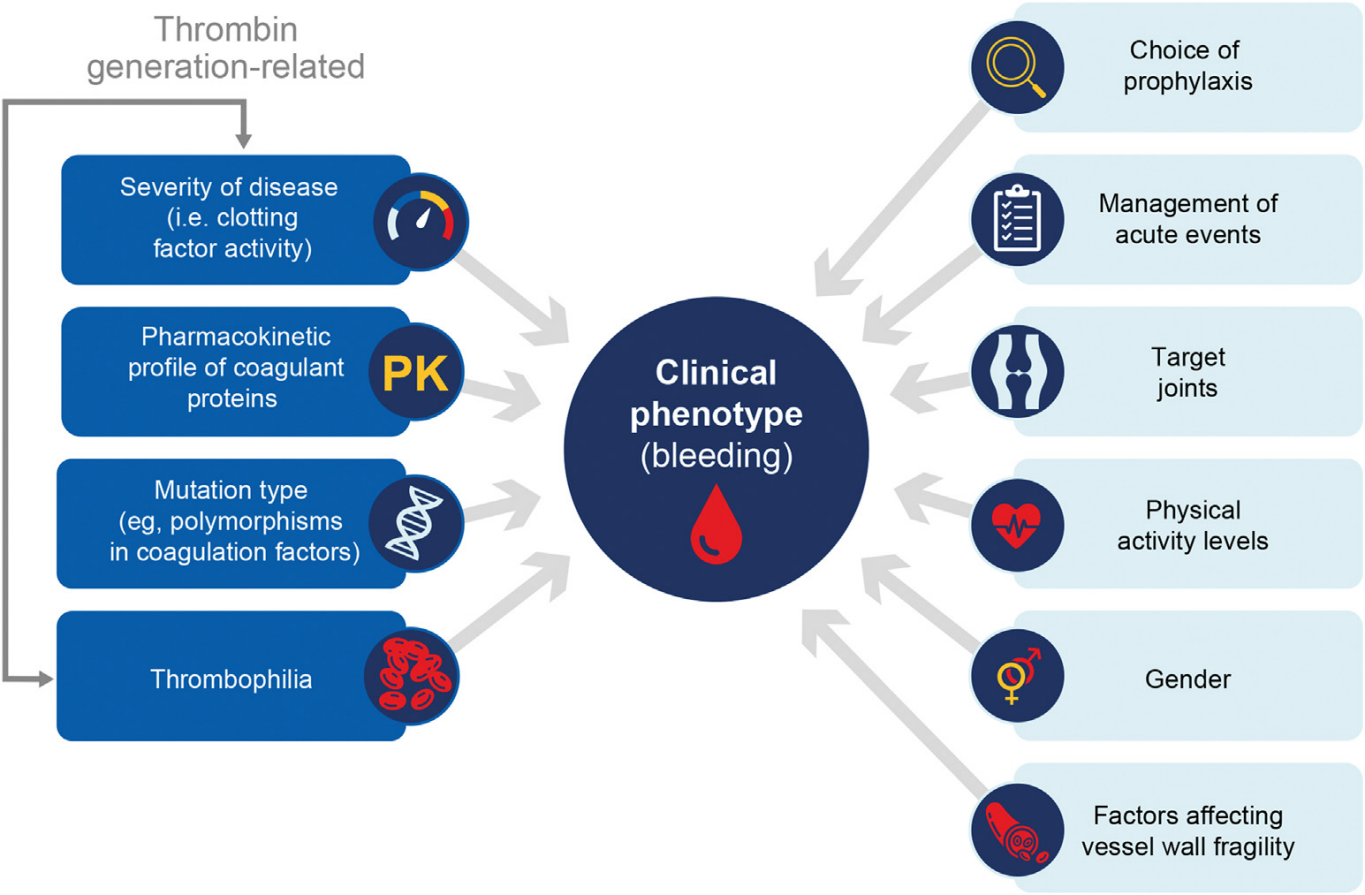
Factors that can impact bleeding phenotype in people with hemophilia

In the early descriptions of hemophilia, severity of the disease (based on plasma clotting factor activity) was generally correlated with the clinical bleeding phenotype (Figure 2) [18]. However, individuals with similar plasma factor may have very different bleeding phenotypes [19]. As many as 10% to 15% of people with severe hemophilia (defined as <1% clotting factor activity) exhibit a relatively mild bleeding phenotype. Some, but not all, of these cases of an unexpectedly mild bleeding tendency can be explained by the coinheritance of a prothrombotic trait [[19], [20], [21]].

A correlation has been found between the bleeding phenotype of people with hemophilia and the pattern of thrombin generation [17]. Brummel-Ziedins et al. [17] evaluated the whole blood TF-initiation profiles for a group of people with mild, moderate, or severe hemophilia, classified using activated partial thromboplastin time (aPTT) parameters, who had varying clinical bleeding phenotypes. When thrombin generation parameters were compared with the bleeding phenotype, the maximum thrombin concentration attained and maximum rate of thrombin generation were significantly different (*P* < .001) between bleeding score groups (group 1: bleeding score of 0 with mild complications; group 2: bleeding score of >0 to ≤9.6 with moderate complications; group 3: bleeding score of >9.6 with most severe complications) [17]. Individuals with increased thrombin generation were protected from bleeding, whereas lower thrombin generation correlated with an increase in clinical bleeding complications [17]. These data suggest that *in vitro* quantitative estimates of thrombin generation may be a useful parameter for predicting and classifying clinical bleeding tendency in hemophilia to aid clinical decision-making [17,22].

## 4 Measurement and Monitoring of Thrombin Generation: Global Assessment of Coagulation

Traditional coagulation measurements, such as prothrombin time and aPTT, can only provide information about the clotting time during the initiation phase of the coagulation process, and the end point of these tests occurs after the formation of only approximately 5% of total thrombin. After the initiation phase, thrombin and fibrin are still being generated [23]. Therefore, these traditional methods are not entirely representative of the complete clot formation process [23], compared with global coagulation assays focused on thrombin generation, which are increasingly used to assess patient bleeding phenotypes and treatment efficacy in bleeding disorders [1].

**Figure 3:**
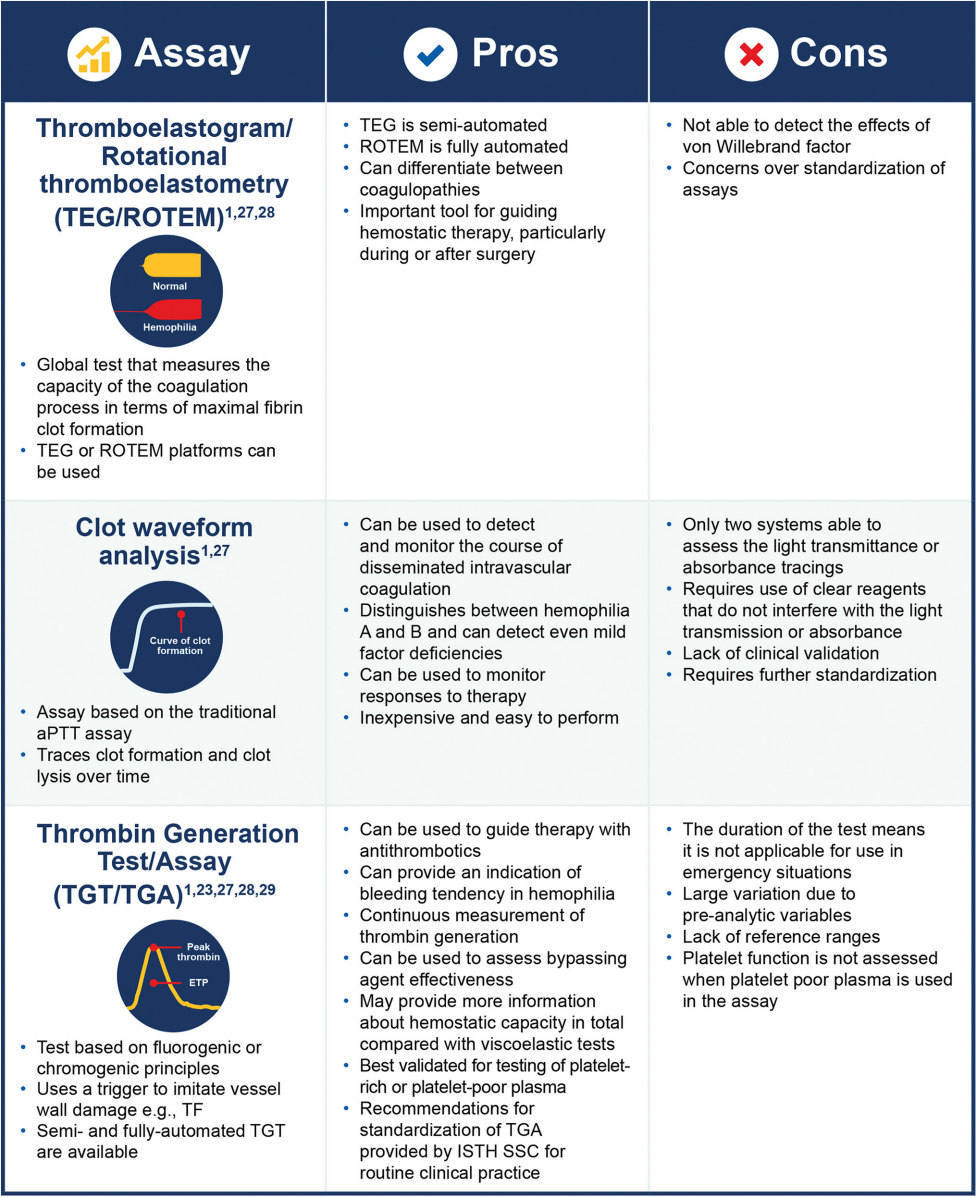
Global coagulation assays: pros and cons. aPTT, activated partial thromboplastin time; F, factor; ISTH SSC, International Society on Thrombosis and Haemostasis International Society on Thrombosis and Haemostasis; ROTEM, rotational thromboelastometry; TEG, thromboelastography; TF, tissue factor; TGA, thrombin generation assay

The most common global assays include thromboelastography (TEG)/rotational thromboelastometry (ROTEM) [24] and aPTT clot waveform analysis, which measure fibrin clot formation, and thrombin generation assays (TGAs), which continuously measure the level of active thrombin in the sample, that results from the simultaneous generation and inhibition of thrombin (Figure 3) [1,9,[25], [26], [27], [28], [29]].

TEG and ROTEM are known as viscoelastic tests and are now automated or semiautomated [27]. Both tests are able to effectively measure the capacity of the coagulation process in terms of maximal fibrin clot formation over time [25,30] and have been shown to be useful for detection and treatment of coagulopathy in trauma care, cardiac surgery, and liver transplantation [27].

Clot wave form analysis is a global assay based on the traditional aPTT assay [1]. It traces clot formation and clot lysis against time and provides information on the velocity of coagulation and fibrinolysis [1,30]. The test has several clinical applications, including monitoring the course of disseminated intravascular coagulation, helping distinguish between people with hemophilia A or B, providing information about the clinical bleeding phenotype of people with hemophilia, and monitoring treatment of people with hemophilia who have been treated with factor concentrates or bypassing agents [1,30]. Goldenberg et al. [31] evaluated the sensitivity of the Clot Formation and Lysis (CloFAL) global assay for FVIII deficiency and found that people with severe hemophilia A showed considerable heterogeneity in CloFAL waveforms despite a similarly decreased coagulation index: the CloFAL assay showed a marked increase in coagulability 30 minutes following FVIII infusion, although in each case, the profile of coagulative response to FVIII infusion determined by the CloFAL assay coagulation index differed qualitatively from that measured by FVIII activity.

The use of a thrombin generation test/TGA in hemophilia was first described by Macfarlane and Biggs [32] in 1953, who demonstrated its utility in monitoring patients with hemophilia. The groundwork for the development of the currently available TGAs was conducted using a chromogenic substrate [30,33]. However, more sensitive fluorogenic substrates are now usually preferred [34]. This is due to not only the increased sensitivity of fluorogenic substrates but also their ability to overcome other limitations of chromogenic substrates. Specifically, chromogenic substrates do not perform well when used in a turbid suspension such as platelet-rich plasma (PRP), due to the interference of turbidity with optical density measurements [23,34]. Thrombin generation parameters measured by TGA include the following: lag time, defined as the time from starting the reaction until thrombin is first generated; peak thrombin, which correlates to the maximum level of thrombin activity achieved in the sample and reflects the thrombin burst during the propagation phase of the coagulation process; time to peak, which corresponds to the time until the maximum amount of thrombin is formed and should be prolonged or shortened in conditions associated with hypo- or hypercoagulability, respectively; and the area under the curve, also known as endogenous thrombin potential (ETP); which reflects the total amount of thrombin generated [34,35].

TGAs are primarily used as a research tool but have been found to be useful in guiding therapy with antithrombotics in patients with arterial thrombosis and for describing bleeding tendency in people with hemophilia [28]. Hemophilia A and B are both characterized by reduced peak thrombin generation, reduced ETP, and prolonged time to peak [12]. Assessment of these parameters by global coagulation assays has been found to provide a reasonable surrogate of an individuals’ hemostatic status [36]. TGAs may provide more information on hemostatic capacity overall than viscoelastic tests because generation of thrombin does not stop when the fibrin clot has formed [23].

TGAs can identify people who have severe hemophilia with a mild bleeding phenotype [37] and those who have a severe bleeding tendency who may require prophylaxis [38]. However, TGAs are limited in their ability to distinguish people with mild bleeding disorders from normal controls because of the variability in thrombin generation among “normal” individuals [39,40]. Knowledge of bleeding phenotype of an individual in combination with the pharmacokinetic profile of coagulant products can be used to tailor therapy to the patient [41].

A linear relationship has been found to exist between FVIII or FIX levels in people with hemophilia and thrombin generation parameters, showing that TGAs could be used to determine *in vitro* the patient-specific FVIII or FIX level to be reached to effectively normalize their thrombin generation and in turn reduce their bleeding tendency [42]. A comparison has been reported for 3 global assays for monitoring patients with FVIII inhibitors: TEG (viscoelastic measurement of fibrin clot formation), Innovance ETP (an assay of thrombin generation using a chromogenic substrate), and Thrombinoscope (an assay of thrombin generation using a fluorogenic substrate). There was a poor correlation between the assay methods. The assays differed in sensitivity to FVIII inhibitors and exhibited different levels of variability. The authors suggested that Thrombinoscope has the most appropriate sensitivity for monitoring patients with hemophilia and inhibitors [43]. Turecek et al. [44] determined that TGAs could also be used to monitor the *in vivo* thrombin-generating capacity during treatment with bypassing agents, thus providing further evidence that TGAs could have a potential use in the optimization of treatment of people with hemophilia and inhibitors. More importantly, TGA results have been effectively utilized in individually tailoring dosing of bypassing agents for people with hemophilia with inhibitors undergoing invasive procedures [45].

A recent study by Aghighi et al. [46] investigated the value of global assays for measuring and monitoring the coagulation potential of people with hemophilia A and compared them with conventional assays. TEG was found to be most suitable for distinguishing moderate from mild hemophilia A, whereas TGAs and clot waveform analysis were more accurate in identifying people with more severe hemophilia A. Heterogeneity of thrombin potential was observed with each of the global assays, indicating that the amount and rate of thrombin generated was not solely dependent on the amount of FVIII in the samples [29]. Global assays demonstrated differences in the coagulation profile of samples compared with conventional assays and indicated that several factors contribute to the severity of the bleeding phenotype in hemophilia [29]. The correlation between thrombin generation and coagulation potential in people with hemophilia suggested that global assays might also be valuable for monitoring before and after treatment with nonfactor therapies [29,47]. For example, TGAs can be used to support decision-making in patients on emicizumab undergoing major surgery to predict both efficacy and potentially minimize the risk of thrombotic events [43].

## 5 Limitations of TGA in a Clinical Setting

TGA has many potential clinical applications, with potential advantages over almost all other techniques, and is a valuable research tool with the ability to predict precrisis changes in both thrombotic and bleeding situations [23,34]. However, this assay also has limitations that need to be overcome before it can be routinely used in most clinical settings [23]. The current World Federation of Hemophilia guidelines for the management of hemophilia acknowledge that TGAs are increasingly being used to monitor response to treatment and to characterize people with hemophilia, but note that their use is not currently recommended because of a need for more data and a lack of availability in most laboratories [48]. The main limitations of TGA use in clinical practice relate to standardization, and the lack of consensus acceptance of the use of thrombin generation for routine patient management [26]. Intra- and interassay variability has improved substantially over the past few years because of improvements in assay precision; however, a survey of TGAs used in “real-life” conditions has found that although intercenter variability for normal and hypercoagulable plasma samples was acceptable at ≤10%, the variability for heparinized plasma samples was still high at 56% [29]. Another important limitation of TGAs is that they are not able to effectively detect changes in the endothelium [23].

To improve standardization, in 2017, an International Society on Thrombosis and Haemostasis Scientific and Standardization Committee proposed preanalytical and analytical recommendations for measuring thrombin generation in the specific setting of hemophilia, which included standardized conditions for blood collection, transport of samples, sample preparation, use of reagents, and temperature of plasma samples [29]. These recommendations suggest that blood for a TGA should be collected by direct venipuncture into a citrate anticoagulated tube, that blood samples should be transported by hand-carrier transport rather than pneumatic tubes to maintain integrity of the samples, and that they should be processed as quickly as possible (ideally within 1 hour of collection) [29]. TF and/or phospholipids are typically used to trigger thrombin generation, and the results of TGAs are highly dependent on the source and concentration of the trigger [29,34]. Use of a standardized trigger reagent is, therefore, recommended to reduce interlaboratory variability. However, there is currently no international reference standard for TF, which is substantially impeding standardization. The use of a reference plasma run in the same experiment to normalize the results has also been found to significantly improve intercenter variability of a TGA and to reduce the importance of temperature variability on the reproducibility of the assay [23,29].

Assay performance can also be affected by the type of sample tested (whole blood vs platelet poor plasma [PPP] vs PRP) [23,36]. For example, for TGAs, PPP is often the most convenient type of sample tested, whereas for viscoelastic methods, the preferred sample is whole blood [36]. Use of PPP or PRP involves time-consuming preanalytical steps that prevent the use of TGAs for rapid testing [23]. There are varying limitations and advantages to each blood fraction, which can translate to difficulties in clinical practice in monitoring the overall effects of the various therapeutic approaches for hemophilia, including nonfactor therapies [36]. A study by Kizilocak et al. [49] found that when activated prothrombin complex concentrate (APCC) was spiked into simulated hemophilia inhibitor plasma with emicizumab *in vitro*, the thrombin generation measurements were significantly different to when APCC was administered to the same patients *in vivo*, suggesting that *in vitro* spiking studies of APCC and emicizumab using thrombin generation may not be valid in this setting.

Standardization and quality assurance of thrombin generation testing and viscoelastic testing can also be impacted by variation in operating procedures of individual practices and laboratories [50]. This should be considered when comparing practical data, and it emphasizes the importance of knowing and understanding the technical details of how individual assays have been performed by other clinical trials and real-world studies [26]. Manual, semiautomated, and fully automated methods are now available to measure thrombin generation. The use of fully automated analyzers has lowered imprecision, including repeatability and reproducibility, to currently accepted laboratory standards of performance for routine assays [34]. However, there is still a need for studies involving larger sets of patients with the use of standardized regents and automated systems to help support the adoption of TGAs in clinical practice [26]. Recently, there has been an increase in publications on TGAs and related testing technologies, which is an indicator of the more widespread acceptance of this assay [34].

## 6 Generating Sufficient Thrombin as the Goal for Effective Hemophilia Treatment

Standard of care for people with hemophilia is prophylaxis, which aims to achieve no spontaneous bleeding [48]. In the past, prophylaxis and the concept of an optimal trough level were viewed in terms of the activity levels of replacement coagulation factor [22]. The World Federation of Hemophilia has proposed a new definition for prophylaxis based on outcomes rather than doses of therapeutic products or time for initiation of treatment regimen. This defines prophylaxis as the regular administration of hemostatic agent/agents with the goal of preventing bleeding in people with hemophilia, while allowing them to lead active lives and achieve quality of life comparable with people without hemophilia [48].

Similarly, the Kreuth V initiative (European consensus proposals) suggests that with increased treatment options, appropriate instruments need to be developed to personalize treatment regimens for all people with hemophilia A and B and recommends that individualization of prophylaxis is the best strategy to improve quality of life, with an ambitious goal of zero bleeding in the near future [51]. As previously discussed, it is also known that the bleeding phenotype does not necessarily correlate with factor level and that examining thrombin generation may be more predictive of bleeding tendency [20]. Thus, it is recommended that coagulation factor activity level should not be the only parameter evaluated, and other parameters, such as thrombin generation, should be used to assess treatment efficacy and to optimize patient outcomes [9].

**Figure 4:**
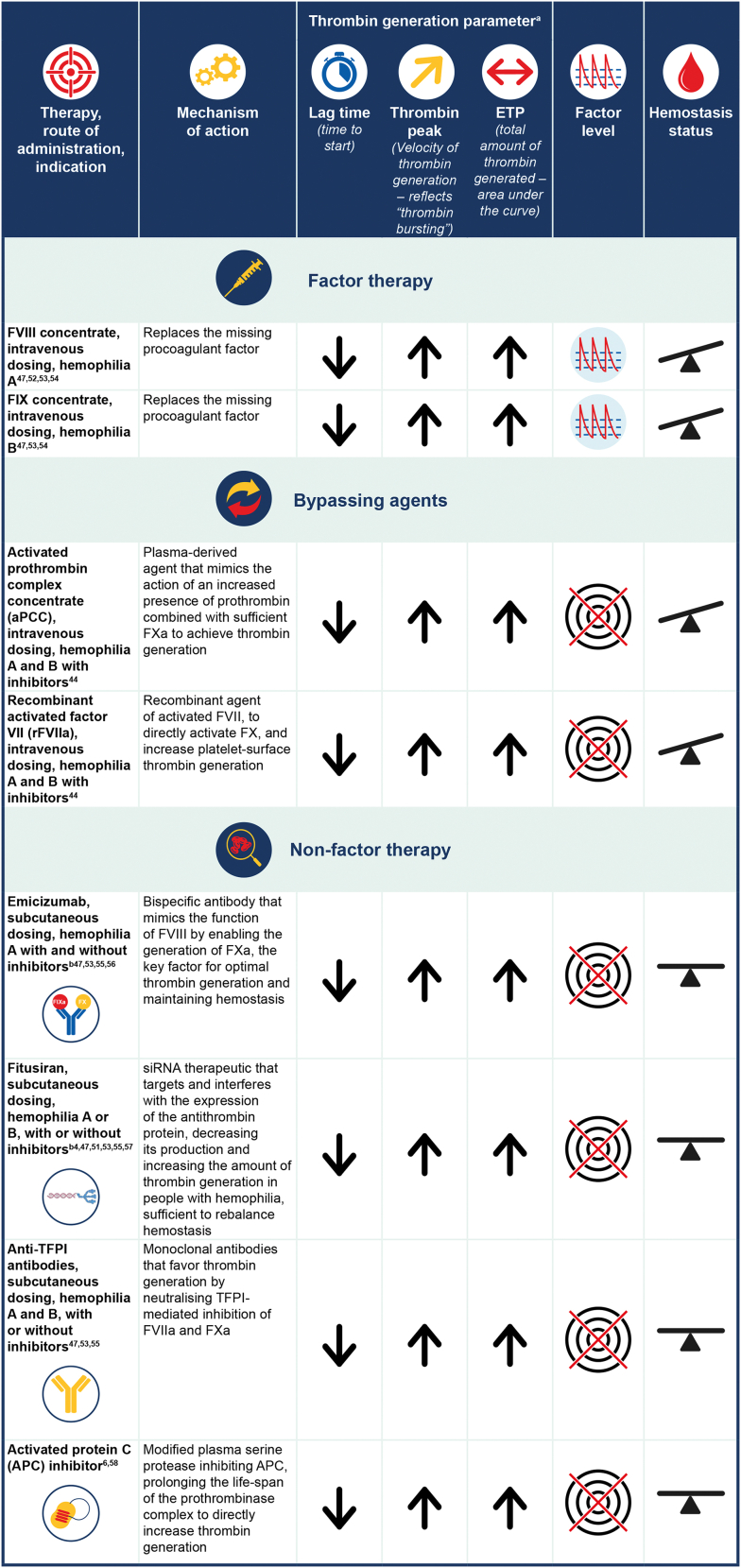
Comparison of parameters related to thrombin generation with different therapeutic approaches (factor vs nonfactor). The impact of the different therapies on the thrombin generation parameters is based on a general scheme rather than evidence-based observations and it should be noted that relative shortening or increasing of lag time or increases in thrombin peak/ETP may depend on the type of TGA and reagent composition being used. = unstable hemostasis (due to peaks and troughs in factor levels);  = very stable hemostasis (with no peaks and troughs in factor levels);  = peaks and troughs in factor levels;  = no direct impact on factor levels (activated prothrombin complex concentrate mimics prothrombin to enhance thrombin generation, rFVIIa targets and enhances thrombin generation, and nonfactor therapies impact on and target stable hemostasis rather than factor levels). ETP, endogenous thrombin potential; F, factor; TFPI, tissue factor pathway inhibitor; TGA, thrombin generation assay. ^a^Postinfusion values. ^b^Time to peak increased after dose after reaching steady state after 4 weeks of loading doses and in the maintenance phase of treatment [52]

All approved and investigational therapies for hemophilia ultimately enhance the generation of thrombin with the aim of restoring effective hemostasis to prevent and control bleeding [1]. The impact of different treatments on thrombin generation in people with hemophilia has been assessed (Figure 4) [1,6,44,47,[51], [52], [53], [54], [55], [56], [57], [58], [59]].

**Figure 5:**
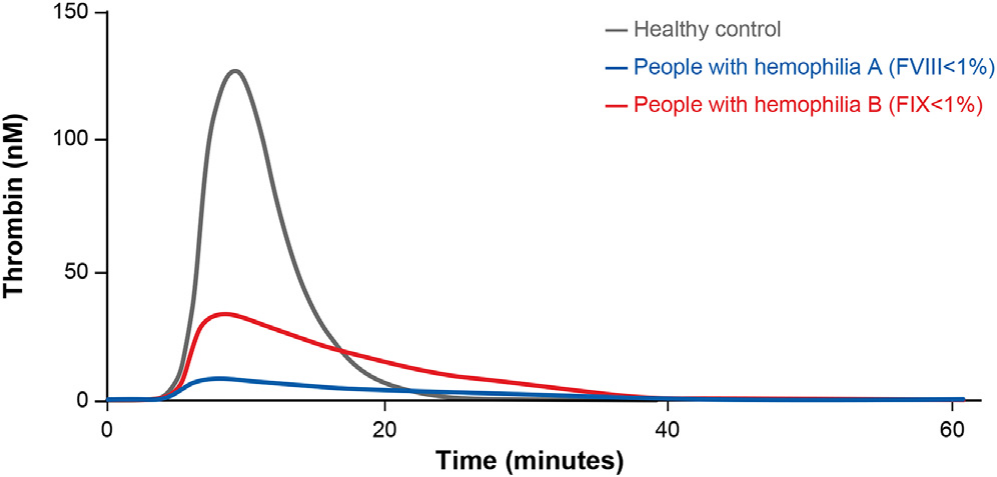
Thrombin generation is greatly reduced in people with hemophilia A and B. Global coagulation assays reveal parameters including peak thrombin and endogenous thrombin potential are reduced in hemophilia. The figure is an example of a representative thrombogram of people with hemophilia A or B vs healthy controls

In a study of 25 people with hemophilia A, Valke et al. [53] showed that each participant had a distinctive thrombin generation response when treated with a single bolus of FVIII concentrate, indicating the importance of determining the thrombin generation profile to prevent under- or overdosing of FVIII. This study adds to the evidence that multiple parameters of thrombin generation are affected in people with hemophilia, including pharmacodynamic factors such as thrombin peak height and thrombin potential, which reflect the hemostatic balance and dynamic, and help tailor treatment to prevent spontaneous bleeds and arthropathy (Figure 5) [36,53,60].

One of the greatest challenges in people treated with FVIII or FIX replacement products is the development of neutralizing antibodies or inhibitors [61]. Inhibitor development occurs in 20% to 35% of people with hemophilia A [62] and up to 3% to 5% of people with hemophilia B [63]. Luna-Zaizar et al. [64] found that TGA could be used to predict individual treatment response to FVIII or APCC therapy in people with severe hemophilia A with high-titer inhibitors [38]. Thrombin generation capacity was found to correlate with the inhibition pattern of antibodies to FVIII clotting activity and could be used to help predict the clinical response to FVIII [64].

People with hemophilia who develop inhibitory anti-FVIII or anti-FIX antibodies rely on bypassing agents such as recombinant activated FVII (rFVIIa) or APCC [62,65]. Overall, in people with hemophilia and inhibitors, rFVIIa or APCC have been shown to increase thrombin generation potential in a dose-dependent manner when given separately, and when combined, the interaction between the 2 products is additive or even synergistic [44,65,66]. These agents aim to boost thrombin generation in plasma to the normal range rather than restoring the missing factors (FVIII or FIX). Therefore, common clinical coagulation assays are not reflective of their hemostatic activity [44,67]. Individual variability in response and a lack of readily available and reliable laboratory methods to predict and monitor their *in vivo* thrombin-generating capacity makes the optimal use of bypassing agents difficult [61,67]. However, as referred to earlier, the global coagulation assays, such as TGA, may be helpful for predicting the response and assessing the effects of bypassing therapy [62,67].

Clinical evidence suggests that nonfactor therapies improve hemostasis in people with hemophilia irrespective of inhibitor status, although breakthrough bleeding may occur and will still require treatment with factor concentrates or bypassing agents [4,54,68,69]. Nonfactor therapies act by enhancing procoagulants (ie, humanized anti-FIXa/FX bispecific antibody—emicizumab, and next-generation FVIIIa mimetic, Mim8), by inhibiting anticoagulant pathways (ie, anti-antithrombin siRNA therapy such as fitusiran, and anti-TFPI antibodies such as concizumab or marstacimab), or by targeting APC inhibiting pathways [47,61,70]. Ideally, nonfactor therapies are being dosed at an equivalent hemostatic level to prevent pathologic bleeding; and it has been found that weekly to monthly subcutaneous delivery of nonfactor therapies allows very stable hemostasis to be achieved but with no factor level [55]. It should be noted, however, that registration trials for nonfactor therapies use a fixed dose without dose adjustment by laboratory testing, which may limit the potential for personalization of prophylaxis with these agents [71].

Emicizumab prophylaxis is given subcutaneously and is currently licensed for the reduction of bleeding events in people with congenital hemophilia A with and without inhibitors [68]. Fitusiran is an investigational, subcutaneous, prophylactically administered siRNA therapeutic, which targets the anticoagulant antithrombin and restores thrombin generation sufficient to rebalance hemostasis in people with hemophilia A or B, with or without inhibitors. Clinical trial data show that bleeding phenotype is improved in patients receiving fitusiran prophylaxis [54,72,73]. Anti-TFPI therapy is under investigation for the treatment of hemophilia A and B, irrespective of inhibitor status, and clinical data have indicated an increase in thrombin generation and a reduced bleeding tendency in patients treated with anti-TFPI therapy [69]. APC-specific serpins have been designed to rescue thrombin generation *in vitro* and restore hemostasis in mouse models, offering a potential alternative novel treatment [6].

A remaining challenge in the treatment of hemophilia is optimizing hemostasis while mitigating the risk of thrombosis. If factor levels are above normal physiological levels (>150%), the risk of thrombosis is increased. This is a key consideration when determining a safe therapeutic range for factor levels in people with hemophilia receiving prophylactic therapy [74]. Prophylaxis dosing with factor replacement increases the trough level of coagulation factor without significantly increasing the peak level to prevent bleeds and improve outcomes. It might be necessary to increase the frequency of dosing or increase the dose of the factor replacement therapy, which in theory increases the risk of thrombosis inherent with these treatments, more notably, if there are comorbidities [22].

Safety concerns have also been raised around thrombotic risk with nonfactor therapies especially when combining therapies with factor concentrates or bypassing agents [4,68]. The occurrence of thrombotic complications during prophylactic treatment with emicizumab, fitusiran, and concizumab has led to modified recommendations for treatment of breakthrough bleeding events [47,69,75]. New recommendations to mitigate the risk of thrombosis suggest using the lowest doses and shortest duration of approved clotting factor concentrates and bypassing agents, as well as limiting the dose of APCC especially in patients receiving emicizumab [61,68]. More research is needed to determine the mechanisms of thromboembolism in patients treated with nonfactor therapies [76].

## 7 Conclusion

Thrombin plays an active and essential role in multiple steps within the coagulation process. The pattern of thrombin generation has been found to have an impact on the physicochemical properties of the resulting clot, including its structure, mechanical stability, and susceptibility to fibrinolysis. Hemophilia is the result of a deficiency of FVIII or FIX leading to reduced thrombin generation. The disease is characterized by greatly reduced peak thrombin generation, reduced ETP, and prolonged time to peak when assessed via TGAs. Thrombin generation may be an accurate marker of bleeding phenotype. The goal of all hemophilia therapies, either by replacing or mimicking the missing procoagulant or by rebalancing through lowering anticoagulants, is to restore sufficient thrombin generation to achieve effective hemostasis. Measurements of thrombin generation have promising utility in monitoring of hemostatic potential of patients and efficacy of new and approved therapies by assessing the overall patient profile before and after therapy to help individualize treatment and improve outcomes with the overall goal of achieving zero bleeds.
